# An *In-vitro* Investigation of Swelling Controlled Delivery of Insulin from Egg Albumin Nanocarriers

**Published:** 2016

**Authors:** Swati Mahobia, Jaya Bajpai, Anil Kumar Bajpai

**Affiliations:** Bose Memorial Research Laboratory, Department of Chemistry, Government Autonomous, Science College Jabalpur, (M.P.) - 482001 India

**Keywords:** Egg albumin, Insulin, Nanocarriers, Biopolymers

## Abstract

The aim of the present work was to prepare and characterize biopolymer nanocarriers and evaluate their suitability in possible oral delivery of insulin. The egg albumin biopolymer was used to prepare nanoparticles which were further characterized by Fourier transformed Infrared spectroscopy (FTIR), transmission electron microscopy (TEM), scanning electron microscopy (SEM), zeta potential, Dynamic Light scattering (DLS) and cytotoxicity. From the characterization studies the size of the nanoparticles washemoly found to lie in the range 20-80 nm with surface charge of -23 mV and also offering extremely fair biocompatibility.. The *in-vitro* biocompatibility of the prepared nanocarriers was judged by BSA adsorption test and haemolysis assay. The in vitro release kinetics of the insulin loaded nanoparticles was studied in phosphate buffer saline (PBS) solution, and the influence of various factors such as pH, temperature and simulated physiological fluids was studied on the controlled release of insulin.

## Introduction

Insulin is the hormone that is synthesized by the beta cells of islets of langerhans in the pancreas. It is composed of 51 amino acids arranged into two polypeptide chains which are connected to each other by two disulphide bonds and responsible to regulate the glucose level of blood for the treatment of diabetes mellitus (1). Diabetes is a worldwide disease which is caused due to the malfunctioning of pancreas which does not produce enough insulin and blood glucose level becomes high (2). Thus, diabetic patients have to take frequent injections of insulin daily which is quite painful and uncomfortable for the patient (3). Hence, the oral delivery of insulin becomes the most desirable route of administration (4). However, reliable and effective oral delivery of insulin encounters some barriers in the gastrointestinal (GI) tract that include enzymatic degradation in the GI tract and poor insulin permeability through the GI system (5). The bioavailability of insulin solution orally comes to be less than 1% (6). Such problems in oral delivery of insulin may be overcome by using biopolymer nanocarriers which are biocompatible and biodegradable in nature and provide protection to the insulin from harsh environment of the stomach.

Egg albumin nanocarriers are known to have particle size in the range of 1-100 nm and have exhibited greater intracellular uptake. Moreover, they are available to a greater range of biologic targets due to their smaller size and mobility (7). Nanocarriers have enormous potential to be targeted to the desired site and they facilitate the uptake of insulin and enhance the oral bioavailability also (8). Nanocarriers have been extensively researched as carriers for oral delivery of insulin (9). Biopolymer nanocarriers are of great interest from a pharmaceutical point of view. It has been observed that the biological effect of insulin loaded nanocarriers depends on the amounts of both the insulin and the biopolymer used. The nature of biopolymer strongly influences the nanoparticle size and release profile (10) and similarly the intensity and duration depends on the site of administration. 

Proteins are natural biopolymers and have advantages such as biodegradability, low toxicity, Non-antigenicity, high nutritional value, high stability and good binding capacity towards drug molecules (11). Egg albumin is one of the most promising protein biopolymers and may be proposed as nanocarriers for oral delivery of insulin as they show high stability in solutions of pH ranging from 4 to 9 and thermal stability up to 60 ^o^C. They have preferentially been used as oral drug delivery systems due to their biodegradability, low toxicity, immunogenicity which make them an ideal material for drug delivery (12). Thus, being motivated by the excellent pharmaceutical properties of egg albumin the authors have attempted to design a swelling controlled drug delivery system for insulin and investigated its release profile under varying experimental conditions to optimize the insulin release under *in-vitro* conditions. 

## Material and methods

*Materials*

Insulin (Activity 40 IU/mL) was purchased from Torrent Pharmaceuticals Ltd. Intrad 382 721 Mehsana India. Egg albumin powder was purchased from Sigma Andrich while its crosslinker glutaraldehyde was obtained from Research Lab, Pune, India. All other chemical used were of analytical grade and double distilled water was used throughout the experiments. 

*Preparation of egg albumin nanoparticles*

The microemulsion method was used for the preparation of nanoparticles. The egg albumin powder (3 g) was dissolved in 100 mL of N/50 NaOH solution under magnetic stirring until it was uniformly dispersed. To this suspension 10 mL of toluene was added with continuous stirring for 45 min to produce a stable emulsion. Now the crosslinker (glutaraldehyde) 5.29 mM was added to the above emulsion, the stirring was continued until the formation of very small droplets of emulsified solution. Now 2 drops N/20 H_2_SO_4_ were added to the emulsified solution of egg albumin which cause precipitation of nanoparticles which were centrifuged and washed three times with acetone. The so prepared nanoparticles of egg albumin were dried in hot air oven so that the nanoparticles changed into slightly yellow fine powder. The so prepared powdered nanoparticles were stored in air tight polyethylene bags. 

*Characterization*

The characterization of nanoparticles are usually done by their size, morphology, surface charge and cytotoxicity towards body following techniques like FTIR (Fourier Transform Infra Red), SEM (Scanning Electron Microscopy), TEM (Transmission Electron Microscopy), DLS (Dynamic Light Scattering), Zeta Potential, *in-vitro* cytotoxicity etc.

*Physiochemical Characterization*

*Fourier Transform Infrared Microscopy*

FTIR spectra of the egg albumin nanoparticles were obtained by using an FTIR spectrophotometer. The egg albumin nanoparticle sample preparation for the FTIR characterization was done by grinding the powdered sample with KBr powder in the ratio of 1:100 for 2-3 min to yield a thin and transparent pellet of KBr. The pellets were scanned over a wave number range of 4000 to 400 cm^-1^. The instrument used to measure absorption spectra were FTIR-8400S, Shimadzu Spectrophotometer.

*Scanning Electron Microscopy*

The surface morphology of the egg albumin nanoparticles surfaces was studied by scanning electron microscopy. Here it provides insights into the morphologies of the insulin loaded and unloaded native nanoparticles of albumin (Scanning electron microscope Shimadzu 2011). 

*Transmission Electron Microscopy*

The transmission electron microscopy was used to investigate the morphology and shape of the egg albumin nanoparticles (13). Transmission electron microscopy (TEM) was recorded by Morgagni-268-D transmission electron microscope with an acceleration voltage of 80 KV.

*Dynamic Light Scattering*

The average particle size and agglomeration of the crosslinked nanoparticles was determined by using Dynamic Light Scattering measurement. It is the most popular method to determine particle size which is based on the principal of the Brownian movements of the various molecular sized nanoparticles (14). The formulations were taken in lyophilized form in microcentrifuge tubes, suspended in phosphate buffer, pH 7.4 and introduced in the instrument to read the results. The instrument used was Beckman Coulter Delso Nano C. 

*Zeta Potential Measurements*

Zeta potential is used to determine the surface charge properties of egg albumin nanoparticles. It is well known that the composition of particles and medium of dispersion affect the surface charges of the nanoparticles. The experimental formulations were taken in lyophilized form in 2 mL eppendorf tube and the samples were suspended in phosphate buffer, pH 7.4 and introduced in the instrument following the guideline of the manufacturer. The results were then read. Zeta potential studies were performed with a digital potentiometer (Model No. 118, EI product, Bhopal India).

*Biopharmaceutical characterizations*

*Water sorption capacity*

The swelling behavior of nanoparticles was investigated by a simple gravimetric technique in which the amount of water absorbed is analyzed by weighing the swollen nanoparticles (15). The swelling behavior of particles depends on the nature of both solvent and polymer (16). For the analysis of water sorption capacity of the albumin nanoparticles, pre-weighed (0.1 g) nanoparticles were immersed in 10 mL phosphate buffer saline (pH 7.4) for swelling at room temperature. After predetermined time intervals the particles were filtered and gently pressed between filter papers for the removal of excess solvent and then weighed. The whole process was repeated at the intervals of half an hour continuously till the equilibrium swelling was achieved. The swelling ratio of the nanoparticles was calculated by using the following equation,

$\mathrm{Swelling ratio}=\frac{W_{t}}{W_{0}}$

Where, *Wo* and* Wt* are the weights of dry and swollen nanoparticles at zero and time t, respectively.

*In-vitro blood compatibility*

For the assessment of suitability of insulin loaded nanoparticles in the internal environment of the body, various tests such as BSA adsorption, percent haemolysis, and cytotoxicity tests were performed for the determination of *in-vitro* blood compatibility.

*Protein adsorption*

The BSA adsorption test is used to perform the adsorption of plasma protein onto the surfaces of nanoparticles when they come in contact with the blood. The plasma proteins (bovine serum albumin, fibrinogen etc.) at the interface of the blood and particles get adsorbed due to which the further adhesion of leukocytes, macrophages or platelets leads to the encapsulation of fibrous part. The BSA adsorption process is based on the batch process (17). 

For doing these experiments, the particles were first equilibrated in PBS solution for 24 h, after that they were filtered and immersed in a known volume of protein solution. They are gently shaken for a definite period of time, centrifuged and the supernatant was collected. The remaining concentration of protein in the supernatant was evaluated by taking absorbance by an UV spectrophotometer (Shimadzu, 1800). The amount of protein adsorbed by the egg albumin nanoparticles was calculated by following equation:

$\mathrm{Adsorbed amount}(\mathrm{mg g}-1)=\frac{\left(\mathrm{Co}-\mathrm{Ca}\right)\mathrm{.V}}{M}\times 100$

Where, Co and Ca are the concentrations of protein solution (mg/mL) before and after adsorption, respectively. V is the volume of the BSA solution; M is the mass of the adsorbent (nanoparticles). 

*Haemolysis assay*

Haemolysis assay tests were performed to determine biocompatibility of the nanoparticles when they come in contact with blood cells. It was performed to know exactly what percent of blood cells get ruptured when in-cooperated with biopolymer nanoparticles (18). For this purpose, fresh human blood was collected in the presence of anti-coagulant and for each experiment always fresh blood was used. In a typical experiment, 0.1 g of nanoparticles were equilibrated in normal saline water (0.9% NaCl solution) for 24 h at 37 ^o^C, after that 0.25 mL of fresh human ACD blood was added to it. It is worth mentioning here that if fresh human ACD blood is not available Then, the stored blood diluted with EDTA anticoagulant can also be used (the addition of anticoagulant reduces the degradation of blood). The blood should not be older than a week for better results. The haemolysis was allowed to take place for 20 min and thereafter 20 mL of saline solution was added to the suspension to stop the process of haemolysis. Then, the suspension was incubated for 60 min at 37 ^o^C. Positive and negative controls were obtained by adding 0.025 mL of human ACD blood and saline solution, respectively to 2.0 mL of distilled water. The incubated samples was then centrifuged for 45 min, the supernatant was taken and its absorbance was recorded on the spectrophotometer at 545 nm. The percent haemolysis was evaluated by using following equation.

$\%\mathrm{Hemolysis}=\frac{A\left(\mathrm{testsample}\right)-A(-\mathrm{sample})}{A\left(+\mathrm{sample}\right)-A(-\mathrm{sample})}\times 100$

Where *A *is absorbance of the samples

*In-vitro Cytotoxicity test*

In order to determine *in-vitro* cytotoxicity of the prepared nanoparticles, in brief, a test sample of the nanoparticles, negative control and positive controls in triplicate were placed on sub confluent monolayer of L-929 mouse fibroblast cells. After incubation of cells with test samples at 37 ± 1 ^o^C, for 24 to 26 h, cell cultures were examined microscopically for cellular response around and under the test sample (19).

*Study on In-vitro release experiments*

*Loading of insulin into the egg albumin nanoparticles*

There are two methods generally adopted for the loading of a drug into the nanocarriers. In the first method, the drug solution is mixed with the biopolymer solution at the time of formation of nanoparticles (20) but this concept has some drawbacks as the purification of nanoparticles may destroy the bioactivity of the loaded drug. While in the second method, the nanocarriers are allowed to swell in the drug solution of definite concentration (21). The percent loading of drug in this case is often high. Thus we adopted this method for the loading of insulin. Here known volume of insulin was diluted with appropriate amount of phosphate buffer saline (PBS) solution and then shaken for the proper mixing of insulin and PBS solution. The loading of drug was performed by allowing 0.1 g of nanoparticles to swell in the 10 mL of insulin solution Until equilibrium. Then, the loaded particles were dried at room temperature and the percent loading of insulin was calculated by using given equation,

$\mathrm{\% loading}=\frac{\mathrm{Wd}-\mathrm{W0}}{\mathrm{W0}}\times 100$

Where, W_d_ is the dry weights of loaded nanoparticles and W_o_ is the dry weights of unloaded nanoparticles.

*Release experiments*

The release experiments were performed by shaking loaded nanoparticles in a definite volume of release medium (PBS, 7.4) by maintaining the speed of shaker for predetermined period of Time. After fixed period of time intervals, the suspension were allowed for centrifugation and the supernatants were withdrawn from the solution and analyzed for the remaining concentration of drug using UV-1800, Shimadzu, UV-Visible Spectrophotometer.

*Release Kinetics*

The release kinetics of the insulin from the nanocarriers can be investigated by considering the release of drug by diffusion process as given by Fick’s law. The whole procedure for the release of drug by nanocarriers can be understood by three consecutive steps: the swelling of insulin loaded nanoparticles in the medium, penetration of water molecules into the nanoparticles so that drug gets dissolved, and lastly diffusion of the drug from the bulk of the nanoparticles into the external release medium. The whole process of release kinetics involves the relaxation of polymeric chain and then the diffusion of entrapped drug into the external medium. The release kinetics data were applied into the following equation that is basically derived from the Fick’s law (22),

$\frac{W_{t}}{W_{\infty }}={\mathrm{Kt}}^{n}$

Where W_t_ and W∞ were the amounts of insulin released at time t and at infinite time (equilibrium amount of insulin released), respectively and k is the rate constant, n is the diffusion exponent That is an indicator of the mechanism of drug transport. When n>0.5, it is non- Fickian diffusion and while n = 0.5 represents a Fickian diffusion. The value of n = 1 represents transport mechanism in which the release of insulin from nanocarriers is zero order. It was observed that the release of insulin takes place when nanocarriers come in contact with the fluid. For the evaluation of diffusion constant of the insulin, the following equation was used,

$\frac{W_{t}}{W_{\infty }}=4{\left[\frac{\mathrm{Dt}}{\Pi L^{2}}\right]}^{0.5}$

Where D is the diffusion constant of the insulin and L is the diameter of the nanocarriers.

*Effect of different parameters on the release of insulin*

There are different parameters which affect the release kinetics of insulin. The factors affecting are Composition of albumin and crosslinker, pH, temperature and ionic strength of release medium etc. 

*Chemical stability of drug*

In order to determine the chemical stability of insulin in different release media and pH, the UV spectral study (UV Shimadzu 1800) was performed which involves recording UV spectra of native insulin solution and released fraction at different pH for different time intervals, respectively.

*Statistical Analysis*

All experiments were done at least thrice. The Figures and Table were presented along with their respective error bars and standard deviations, respectively.

## Results and Discussion

*Reaction mechanism of the preparation of egg albumin nanoparticles*

**Figure 1:**
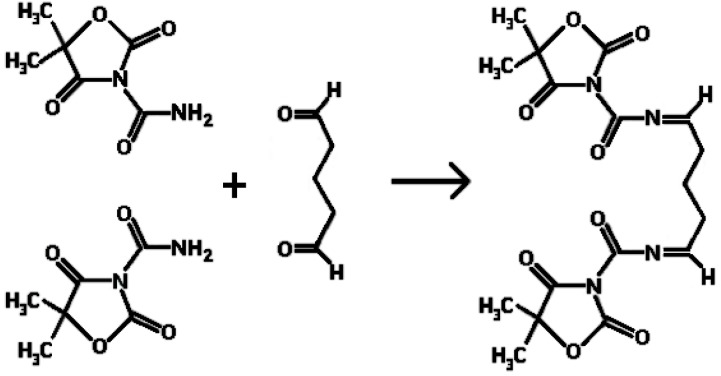
Reaction mechanism for the crosslinking of egg albumin nanoparticles

Figure 1. shows the reaction mechanism of the preparation of egg albumin nanoparticles crosslinked with glutaraldehyde. It is shown that the amide bond of NH_2_ molecule when comes in contact with the carbonyl group of glutaraldehyde, reacts to form the -N=C- bond by losing a molecule of water. In this way the nanoparticles of egg albumin crosslinked with glutaraldehyde are formed.

**Figure 2:**
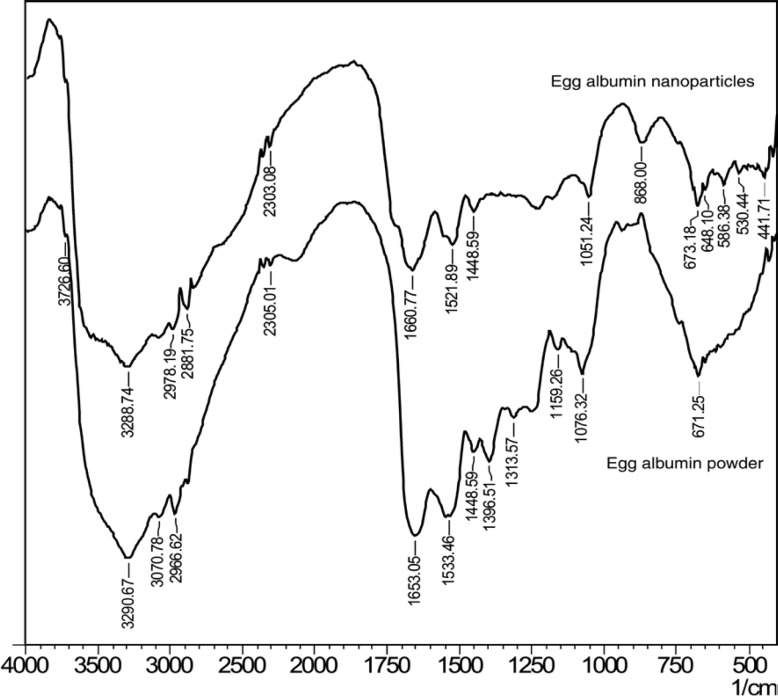
FTIR spectra of (a) Egg albumin powder and (b) Egg albumin nanoparticles

**Figure 3:**
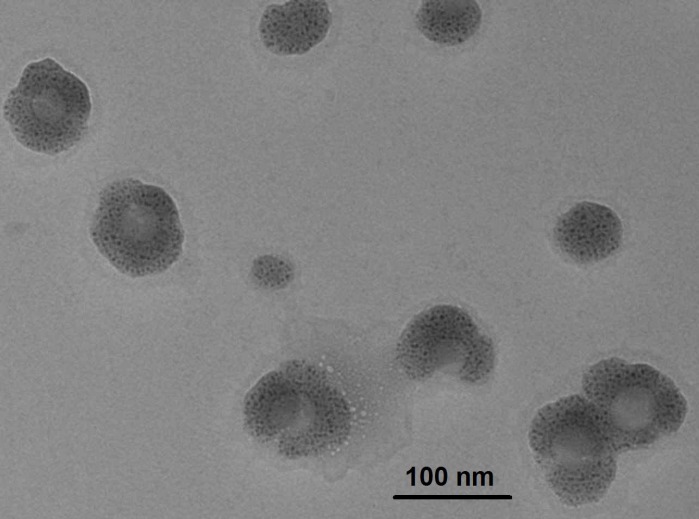
TEM image of egg albumin nanoparticles

**Figure 4.**
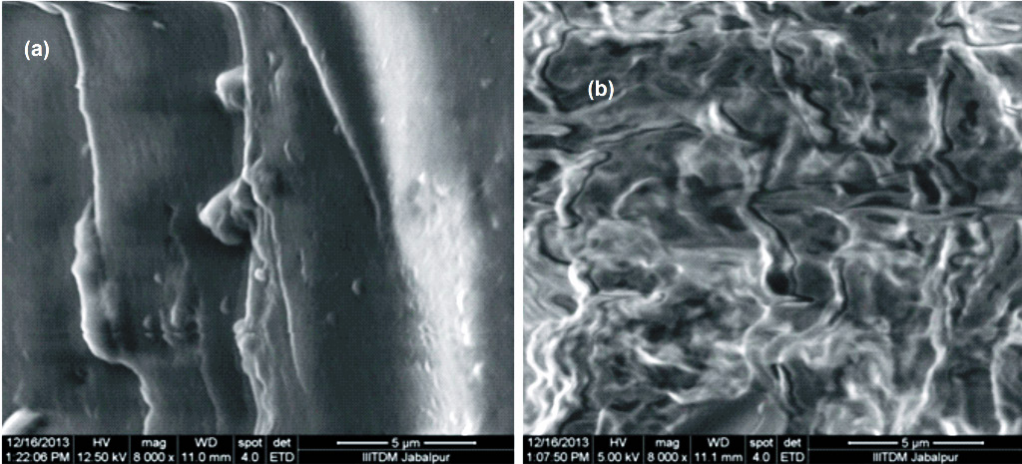
SEM images of egg albumin nanoparticlesInsulin loaded,and (b) unloaded

**Figure 5:**
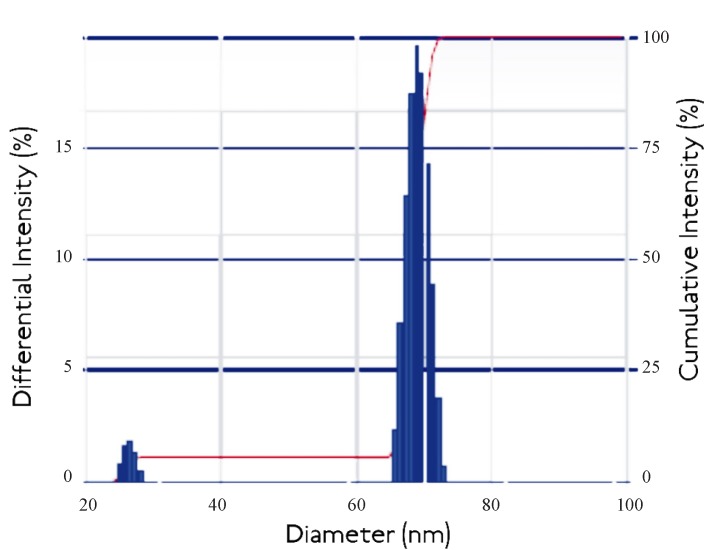
Dynamic Light Scattering curve of egg albumin nanoparticles

**Figure 6:**
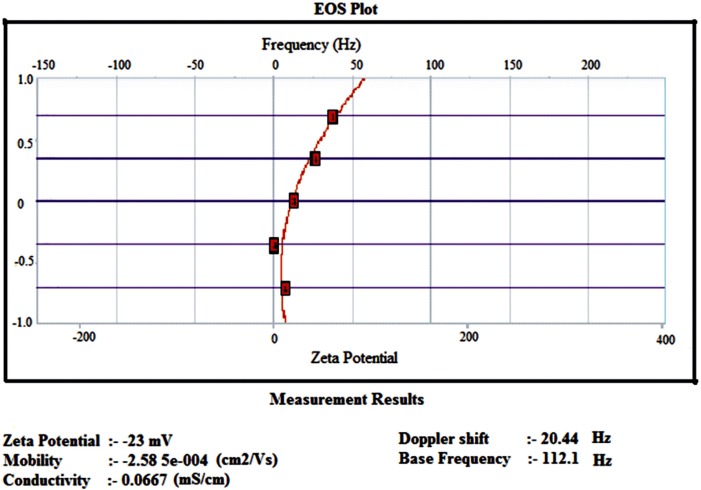
Zeta Potential curve of egg albumin nanoparticles

**Figure 7:**
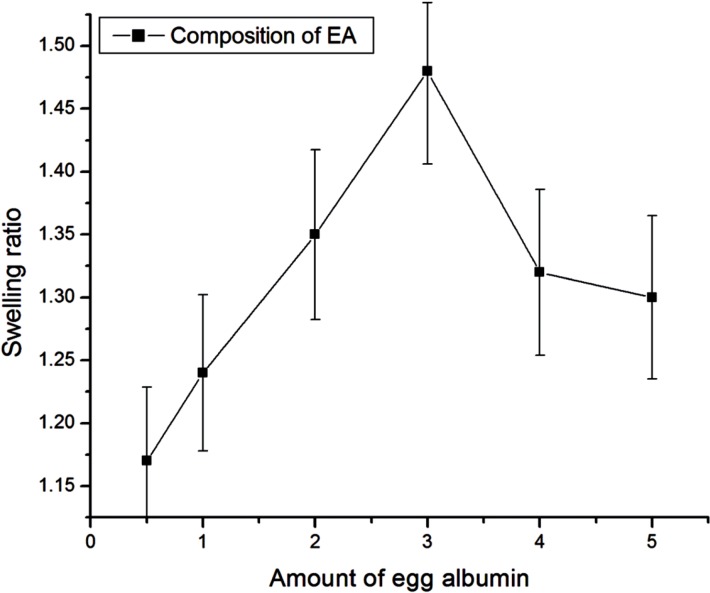
Effect of amount of egg albumin on swelling of nanoparticles

**Figure 8:**
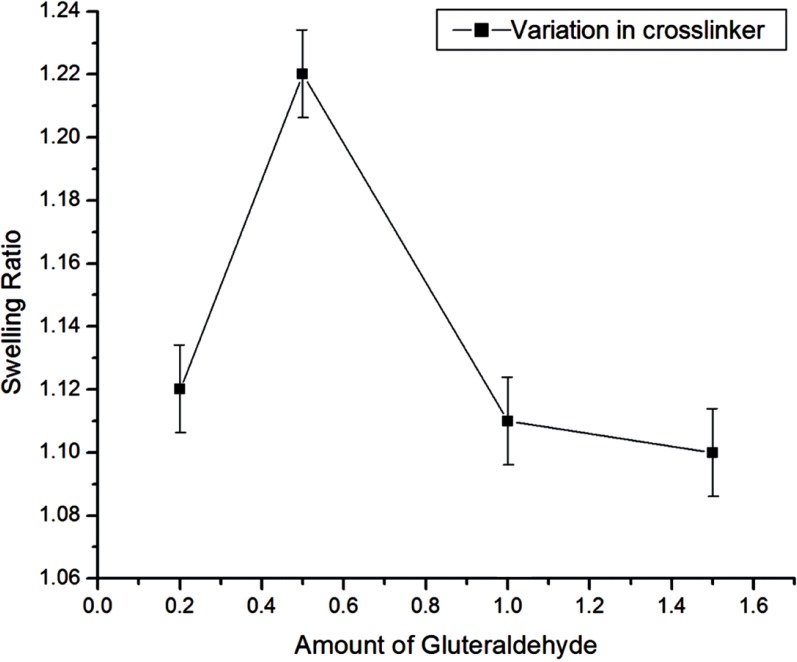
Effect of crosslinker variation on swelling of egg albumin nanoparticles

**Figure 9:**
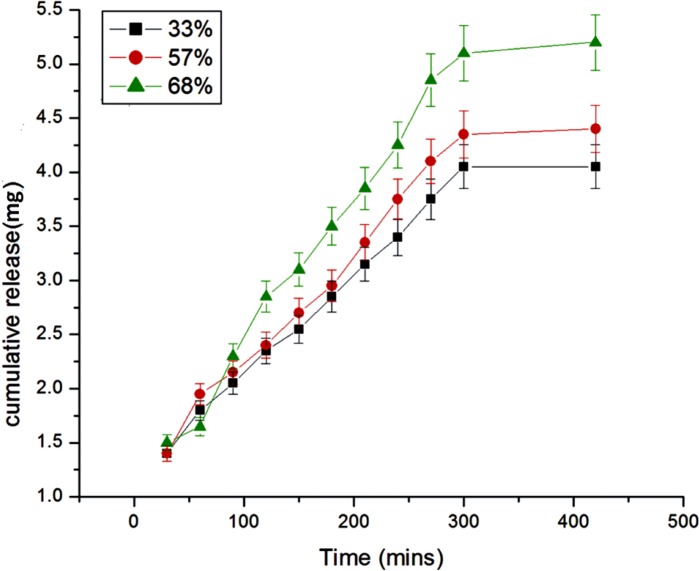
Effect of percent loading on the release of insulin

**Figure 10:**
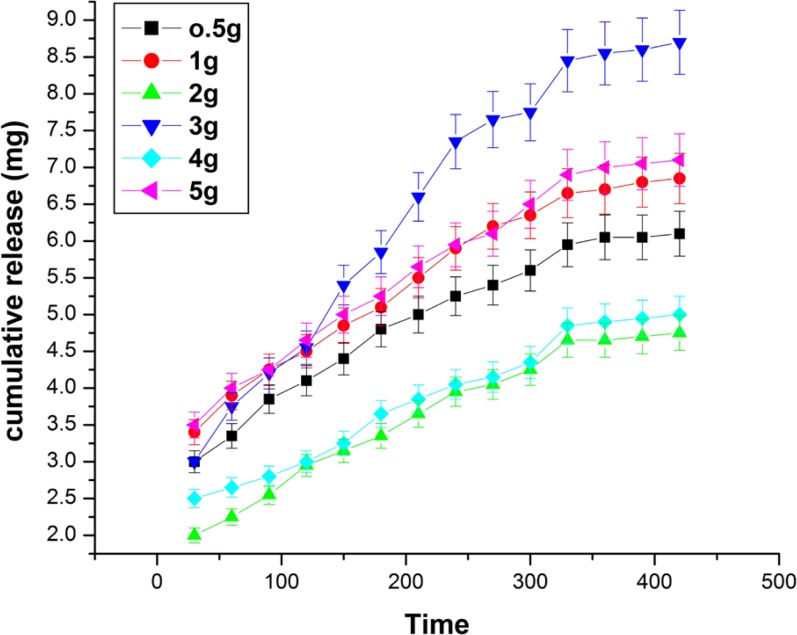
Effect of albumin on the released amount of insulin

**Figure 11:**
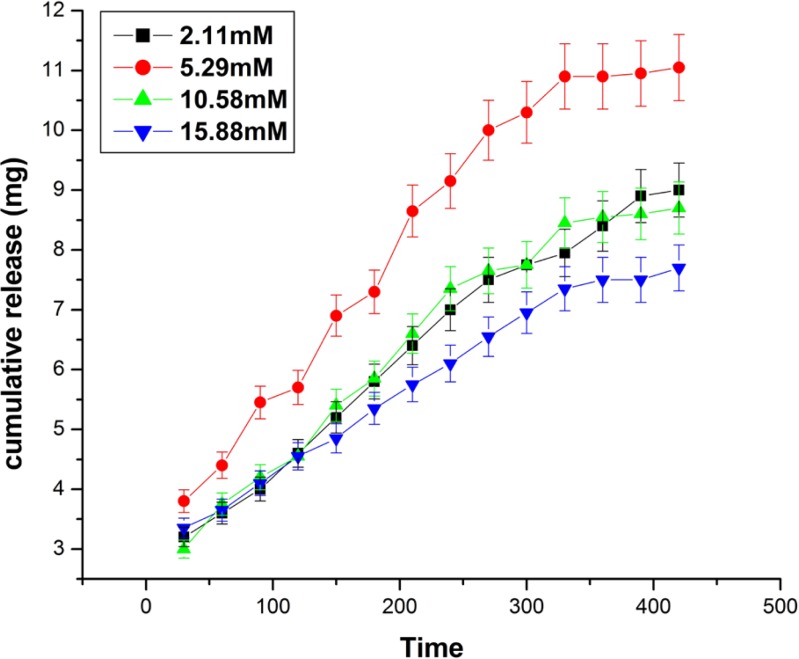
Effect of crosslinker concentrations on the release kinetics of insulin

**Figure 12:**
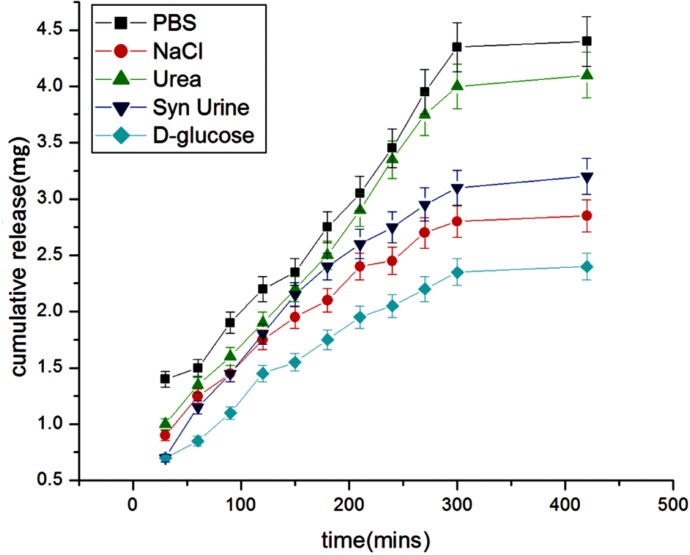
Effect of physiological fluids on the release of insulin

**Figure 13:**
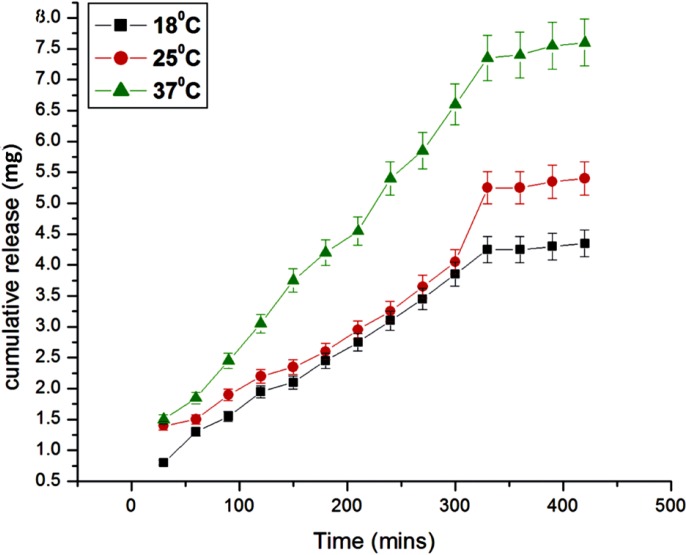
Effect of temperature variation on the release of insulin

**Figure 14:**
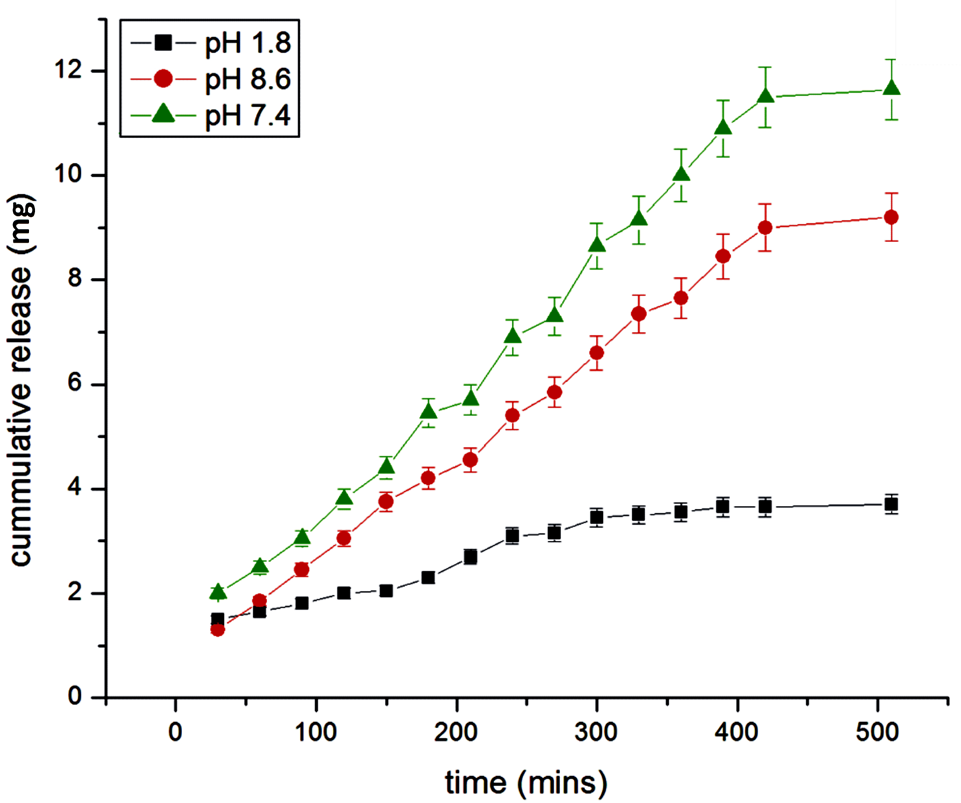
Effect of pH variation on the release of insulin

**Figure 15:**
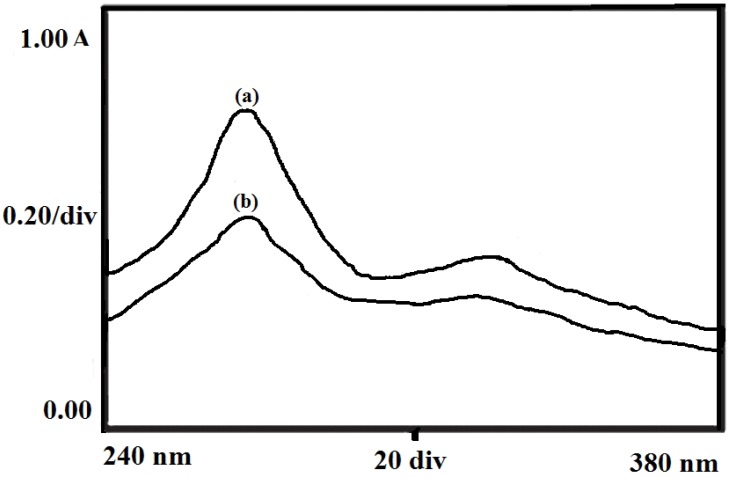
UV spectra showing the chemical stability of insulin in its pure solution (a) and release media (b

**Table 1:** Data showing the biocompatibility parameters with varying composition of egg albumin nanoparticles

S.N.	Glutaraldehyde(mM)	Egg Albumin (g)	Amount of protein adsorbed (mg/g)	Haemolysis (%)
1	5.29	0.5	26.09±0.78	30.05±0.90
2	5.29	1.0	23.04±0.69	29.67±0.89
3	5.29	2.0	22.06±0.66	31.42±0.94
4	5.29	3.0	15.68±0.47	22.68±0.68
5	5.29	4.0	30.01±0.90	33.24±0.99
6	5.29	5.0	28.44±0.85	36.48±1.09
7	2.11	3.0	29.68±0.89	28.04±0.84
8	10.58	3.0	32.08±0.96	32.02±0.96
9	15.88	3.0	38.02±1.14	33.09±0.99

**Table 2.** Quantitative evaluation of in-vitro cytotoxicity reactivity of egg albumin nanoparticles

**1**	**Negative control**	**0**	**None**
**2**	**Positive control**	**4**	**Severe**
**3**	**Sample**	**0**	**None**

**Table 3:** Kinetic analysis of insulin release data summarizing the release exponents and diffusion coefficients under varying experimental conditions

**S.N.**	**Glutaraldehyde** **(mM)**	**Egg Albumin (g)**	**Diffusion ** **Coefficient** **(cm2/min)**	**n**	**Mechanism**	**R** **2**
1	5.29	0.5	0.43 x 10-14	0.70	Non- Fickian	0.98
2	5.29	1	0.46 x 10-14	0.75	Non- Fickian	0.99
3	5.29	2	0.32 x 10-14	0.61	Non- Fickian	0.98
4	5.29	3	0.44 x 10-14	0.62	Non- Fickian	0.96
5	5.29	4	0.37 x 10-14	0.64	Non- Fickian	0.99
6	5.29	5	0.34 x 10-14	0.61	Non- Fickian	0.99
7	2.11	3	0.44 x 10-14	0.84	Non- Fickian	0.99
8	10.58	3	0.53 x 10-14	1.0	Anamalous	0.98
9	15.88	3	0.34 x 10-14	0.5	Fickian	0.98

*FTIR spectroscopy*

FTIR spectroscopy has been used to determine the functional groups of the egg albumin nanoparticles crosslinked with glutaraldehyde. The FTIR spectra of egg albumin powder and egg albumin nanoparticles are shown in Figure 2(a) and (b). respectively which confirms the presence of characteristic groups in the nanoparticles. In the FTIR spectra of the egg albumin powder, the characteristic peaks were observed at 1653 cm^-1^ (–C=O stretching) due to amide I band and 1533 cm^-1^ (C-N stretch with N-H bending mode) due to amide II band which shows the presence of protein (23). Because each of the secondary structure was associated with a characteristic hydrogen bonding pattern between amide C=O and N-H groups, it was expected that each type of secondary structure will give rise to characteristic amide I and II absorptions that allows determination of protein secondary structure by IR spectroscopy. Similarly the egg albumin nanoparticles show peak at1521cm^-1^ which slightly shifts to the lower wavelength due to change in N-H bond.

*TEM analysis*

The TEM technique was used to determine the internal structural morphology and particle size of egg albumin nanoparticles. It is well known that the particles more than 200 nm tend to be eliminated from the organs while smaller size of nanoparticles have larger time to stay inside the body and affecting the oral delivery of insulin.

In the present study, the TEM image of the nanoparticles is shown in Figure 3. which clearly depicts that the particles are almost spherical in shape and are present as aggregates. The size of the particles does not vary significantly from each other and falls within the approximate range of 10 to 30 nm. Thus, the nanoparticles prepared in this study may be of potential use for oral delivery.

*SEM analysis*

SEM is the technique used to determine the external morphology of the nanoparticles and it reveals whether the nanoparticles are smooth or rough in their surfaces. The morphology of the egg albumin nanoparticles were analyzed at the magnification of 8000. It was observed that the nanoparticles were in aggregated form and seems smoothed externally. Figure 4(a)and (b) depicts the images of both the insulin loaded and unloaded particles in which loaded nanoparticles shows coating of insulin onto the surface of nanoparticles. The unloaded particles show aggregation of nanoparticles. 

*Dynamic light scattering measurements*

Dynamic light scattering is an important tool for characterizing the size of nanoparticles in solution. DLS measures the light scattered from a laser that passes through a colloidal solution and by analyzing the modulation of the scattered light intensity as a function of time, the hydrodynamic size of particles and particle agglomerates can be determined. Larger particles will diffuse slower than smaller particles and the DLS instrument measures the time dependence of the scattered light to generate a correlation function that can be mathematically linked to a particle size (24). It was known that the DLS study gives the idea about cumulative intensity and differential intensity which helps in resolving the particle size distribution of polydisperse system. 

Thus the Intensity distribution graph as shown in Figure 5. it was shown that the 95% cumulative intensity of the nanoparticles and 15% differential intensity falls in the range of diameter of about 40-60 nm. The observed results are not consistent with the findings of the TEM studies which also reports the size of nanoaprticles in the range 10 to 30 nm. The observed higher range of Nanoparticles size from DLS measurements may be due to the agglomeration of egg albumin Nanoparticles in the solution. 

*Zeta potential*

Zeta potential analysis is a technique for determining the surface charge of nanoparticles in solution (colloids). Nanoparticles have surface charge that attracts the thin layer of ions of opposite charge to the nanoparticles surface. This double layer of ions travels with the nanoparticles as it diffuses through the solution. The electric potential at the boundary of the double layer is known as the Zeta potential of the particles and has values that typically range from +100 mv to -100 mv (25). The magnitude of the zeta potential is predictive of the colloidal stability. Nanoparticles with zeta potential values greater than +25mv or less than -25mv typically have high degree of stability. Figure 6. shows that insulin loaded nanoparticles had the zeta potential of -23mv which shows the higher stability of nanoparticles.

*Swelling studies*

*Effect of composition of egg albumin*

The effect of the varying amount of egg albumin on water sorption capacity has been investigated by varying it in the range from 0.5g - 5 g. It is well recognized that the swelling behavior of the nanoparticles greatly depends on the amount of polymer present. The swelling studies show that the swelling of albumin nanoparticles increases as its amount increases in the composition up to 3 g and after that the swelling of nanoparticles decreased as shown in Figure 7. The increase in swelling of nanoparticles is due to an increase in hydrophilicity of the particles and hence the water absorption ability of particles increases. Whereas the observed decrease in swelling of nanoparticles beyond a certain amount of egg albumin may be due to the fact that the particles becomes more integrated and compact thus allowing less number of water molecules to enter nanoparticles network.

*Effect of crosslinker variation*

The effect of crosslinker was examined by adding the amount of crosslinker ranging from 2.11 mM to 15.88 mM during the preparation of nanoparticles while keeping the amount of albumin (3g) as constant. In this case, the swelling ratio of particles increases up to 5.29 mM of glutaraldehyde and then decreases upon further increase in crosslinker concentration as shown in Figure 8. The results may be explained due to the fact that up to 5.29 mM of crosslinker, the degree of crosslinking of nanospheres increases and therefore the size of nanospheres gets smaller which results in the increase of internal and external surface area of the particles. Thus, the sorption of water molecules increases. Whereas, by increasing the crosslinker concentration beyond 5.29 mM, the swelling ratio of the nanospheres decreases and this may be attributed to that fact that beyond 5.29 mM of the crosslinker, the nanospheres gets crowded with glutaraldehyde chain in small area, due to which there is less volume left for entrance of the water molecules for the further binding with the nanospheres. Thus the degree of swelling ratio depends on the network of crosslinker within the nanospheres which allows the imbibitions of water molecules in it, and hence it affects the *in-vitro* release of insulin also.

*Release studies*

*Effect of percent loading*

The amount of insulin loaded onto nanoparticles is expected to exert a great influence on the release dynamics of insulin and this has been achieved by loading different percent of insulin onto the egg albumin nanoparticles. The results are presented in Figure 9. which clearly shows that as the percent loading increases from 33 to 68 percent the amount and rate of insulin release also increase. The results are quite usual and may be explained by the fact that with increasing percent loading greater number of insulin molecules will diffuse out from the nanoparticles network and result in larger drug release. The greater release may also be attributed to the fact that higher percent loading causes a faster movement of solvent front into the nanoparticles and results in a larger release of insulin (26).

*Effect of albumin composition*

The effect of albumin variation on the release of insulin has been investigated by varying its amount from 0.5 to 5 g in the feed mixture of the nanoparticles. The release results are shown in Figure 10. which indicate that the cumulative release of insulin increases with increasing amount of albumin from 0.5 g to 3.0 g while beyond 3.0 g the released amount of insulin constantly decreases. The observed results may be explained by the fact that albumin is a hydrophilic biopolymer and its increasing amount in the particles will obviously enhance the hydrophilicity of the nanoparticles and thus released amount of insulin increases (27). However, at higher amount of albumin content, the nanoparticles become more compact and as a consequence less number of water molecules penetrates Into the nanoparticles. This clearly results in a decrease in the amount of insulin released.

*Effect of glutaraldehyde*

The effect of glutaraldehyde variation on the released amount of insulin has been investigated by varying the concentration of glutaraldehyde in the range of 2.11mM to 15.88 mM. The results are shown in Figure 11. which clearly indicates that the cumulative release of insulin increases with increasing concentration of glutaraldehyde from 2.11 mM to 5.29 mM, while beyond it a fall in the released amount of insulin is noticed. The results clearly indicate that increasing concentration of glutaraldehyde results in longer chain lengths of biopolymer, hence the mesh size of the free volume available in between the macromolecular chains increases whereas, beyond 5.29 mM the released amount of the insulin decreases which may be because of the reason that at higher concentration of the glutaraldehyde, the biopolymer chain of nanoparticles becomes largely crowed (28).

*Effect of physiological fluids*

The effect of physiological fluids on the released insulin was examined in different physiological fluids such as urea, synthetic urine, saline water, glucose, PBS. The results are shown in Figure 12. which show that the presence of solutes suppresses the drug release. The reason for the lower release of insulin in these simulated fluids is due to the fact that the presence of salts ions in the release media lowers the osmotic pressure of the nanoparticles (29) which results in a lower swelling of the nanoparticles network and thus causes a lower release of insulin.

*Effect of temperature*

The effect of temperature on the release of insulin from the egg albumin nanoparticles depends on the behavior of nanoparticles towards different temperature. It is known that the release of insulin depends on both the mobility of the biopolymer nanoparticles as well as diffusion of insulin molecules; the effect of temperature on the release of insulin has been investigated by varying the temperature of release media from 15 ^o^C to 37 ^o^C. The results are shown in Figure 13. which show that with the increase in temperature the released amount of insulin also increases. The reason for the observed increase in the release of insulin with increasing temperature is due to fact that the network chains within the nanoparticles undergo faster relaxation due to increase in kinetic energy of molecules which allow large water sorption and hence the release of insulin increases (30).

*Effect of pH*

The effect of pH has been investigated in the range of 1.8-8.6, which was identical to the internal pH of the GI tract. The results are shown in Figure 14. which shows a lesser insulin release in acidic and alkaline medium while showing an optimum release in neutral medium. The release of insulin is directly concerned with the swelling behavior of nanoparticles at different pH. It was found that at pH 1.8, swelling ratio was decreased because most of the carboxyl groups in albumin exist in the form of –COOH in lower pH hence in the nanoparticle network, H-bonding constructed by –COOH groups of albumin led to the stronger interaction between polymer chains. Thus, in acidic medium, the swelling is controlled mainly by the amino group (NH_2_). At higher pH, the carboxylic acid group gets ionized and acquires –COO form. 

Thus, the weak H-bonding interaction between polymer chains and electrostatic repulsion between–COO groups resulted in the higher swelling ratio. Due to this osmotic pressure difference between the internal and external solutions of the polymeric network is balanced by the swelling of the particles. However, under very highly acidic conditions (pH<3), a screening effect of the counter ions, i.e. Cl^−^, shield the charges of the ammonium cations and prevent an efficient repulsion. As a result, a remarkable decrease in equilibrium swelling is observed. Again, the screening effect of the counter ions (Na^+^) limits the swelling at pH>8.5. 

When the ionic strength of the solution is increased, the difference in osmotic pressure between bulk of the particles and the medium decreases which results into lowering of swelling capacity, thus the release of insulin vary with pH (31).

*Drug Activity*

The chemical stability of the insulin was investigated by recording the UV spectra of native insulin molecules in the solution and released insulin from the particles at 272 nm. Figure 15. shows the spectra of native and released insulin which reveals that the two spectra are almost identical thus suggesting for no change in chemical and bioactivity of the insulin following the loading and release of insulin.

*Evaluation of biocompatibility*

The biopolymer to be used as biomaterial are based on various factors such as physiochemical properties, desired functions, nature of physiological environment, adverse effects in case of failure, expected durability and consideration relating to cast and ease of production. But of all, the most important features of the nanoparticles used in drug delivery are its biocompatibility towards body (32). Thus, the assessment of biocompatibility is essential and it has been made on the basis of two *in-vitro* tests, viz. BSA adsorption test and haemolysis assay as discussed below:

*BSA adsorption test*

The biocompatibility of the egg albumin nanoparticles has been investigated by observing the amount of protein adsorbed by the nanoparticles. Protein adsorption on polymer surfaces has great importance in biomedical applications both *In-vitro* and *in-vivo*. It is known that the adsorption of proteins depends on the surface morphology and nature of particles whether it is hydrophilic or hydrophobic or a combination of both. For instance, the rough surfaced particles adsorb more protein while smooth particles do not adsorb much protein. Similarly the hydrophobic nature of particles favors higher amount of protein adsorption while hydrophilic nature of particles allows less protein adsorption (33). 

The results are summarized in Table 1. which indicate that the amount of adsorbed BSA decreases with increasing amount of albumin in the feed mixture of particles. From the SEM studies it may be seen that the external morphology of the particles was smooth and they are hydrophilic in nature also which do not damage the blood cells or the plasma protein which is the main requisites for biocompatibility. It is also clear from the Table 1. that by increasing the amount of crosslinker the water sorption capacity decreases which may favor BSA adsorption.

*Haemolysis assay*

Haemolysis studies are performed to ascertain that whether the nanoparticles are biocompatible or not. Under the haemolysis assay, the nanoparticles of varying composition were allowed to interact with the blood cells and the percent haemolysis was analyzed. The results are shown in Table 1. That indicate that on increasing the amount of albumin the percent of haemolysis decreases while on increasing the amount of glutaraldehyde the percent haemolysis increases which implies the enhanced thrombogenicity. Thus, the results reveal that on changing the composition, hydrophilic nature of nanoparticles favors decrease in extent of haemolysis. The hydrophilic nature of the nanoparticles does not allow the blood proteins or blood plasma to be adsorbed on to surfaces and hence does not damage the blood cells. Hence,the percent haemolysis decreases with increase in composition of egg albumin and thus improves the blood compatible quality of the nanoparticles.

*In-vitro cytotoxicity*

*In-vitro* cytotoxicity are decided on the bases of grades designated as 0, 1, 2, 3 and 4 representing none, slight, mild, moderate and severe, respectively shows the zone of lyses, vacuolization, detachment and membrane disintegration respectively. The results are summarized in Table 2 which represent quantitative evaluation of reactivity for negative controls, positive controls and test samples shown through the microscopic observations. It was observed that the test samples showed none reactivity of fibroblasts cells even after 24 h of contact. The numerical grade more than 2 is considered cytotoxic. In the present study, the biopolymer nanoparticles achieved a reactivity grade not more than 2, hence the sample was considered non cytotoxic. Negative control shows none cytotoxic reactivity while positive control shows severe cytotoxic reactivity.

*Kinetic analysis of release data*

When the insulin loaded nanoparticles comes in contact with a solvent, relaxation of polymer chains takes place. This allows the amount of solvent to be entered into the particles and hence the diffusion of insulin into the external medium takes place by crossing the swollen polymer network of the nanoparticles. The relaxation of polymer chains and diffusion of insulin molecules determine the type of release mechanism being followed by the insulin molecule. It has been followed by Higuchi equation that if n = 0.5, the release is diffusion controlled, whenthe value of n is in between 0.5-1.0, the release is non Fickian, and when the value comes n = 1.0 (case II), the mechanism becomes anomalous. In some cases n has been found to exceed 1.0 and the mechanism is termed as super case II. The value of diffusion coefficient (D) and release exponent (n) have been calculated as described above and are summarized in Table 3.

## Conclusions

In the present work, the nanoparticles of egg albumin prepared by micro emulsion crosslinking method were examined for swelling studies and insulin release kinetics. It was clearly observed that the water sorption capacity was influenced by the chemical composition of the nanoparticles and the release kinetics depends on various factors such as temperature, pH, simulated physiological fluids, variation in composition of albumin and glutaraldehyde. The biocompatibility of the nanoparticles were confirmed by BSA adsorption, haemolysis assay and while its immunogenicity was investigated through *in-vitro* cytotoxicity. It was found that the release of insulin increases with increase in temperature and similarly the effect of pH also influenced the release of insulin in which it decreased in acidic and alkaline medium while increased in neutral pH. The variation in release of insulin was also observed in case of simulated fluids in which the maximum release was observed in PBS while it goes down in other physiological fluids such as saline water, urea, synthetic urine and D-glucose solution. It was also confirmed that the release of insulin increases with increase in albumin content of nanoparticles to some extent and then decreases. Furthermore, in case of crosslinker it decreased with increase in glutaraldehyde.

The surface morphology, shape, size and surface charge of the nanoparticles was examined by various analytical techniques such as FTIR spectroscopy, SEM, TEM, DLS and Zeta Potential measurements which clearly confirm the crosslinking of nanoparticles. The characteristic functional groups of egg albumin and their crosslinking with glutaraldehyde were clearly determined by FTIR spectra. Similarly other techniques help in determining the shape and size of nanoparticles. Through *in-vitro* cytotoxicity test, it was confirmed that the particles have no cytotoxicity at all.

## References

[1] Beals J, Brader M, De Felippis M, Kovach P, Crommelin D, Sindelar R (2002). Insulin. Pharmaceutical biotechnology.

[2] Kumar PJ, Clark M (2002). Textbook of Clinical Medicine.

[3] Zambanini A, Newson RB, Maisey M, Feher MD (1999). Injection related anxiety in insulin-treated diabetes. Diabetes. Res. Clin. Pract.

[4] Gowthamarajan K, Kulkarni G (2004). Oral insulin: fact or fiction?. Resonance.

[5] Khafagy ES, Morishita M, Onuki Y, Takayama K (2007). Current challenges in non-invasive insulin delivery systems: a comparative review. Adv. Drug. Deliv. Rev.

[6] Lowman AM, Morishita M, Kajita M, Nagai T, Peppas NA (1999). Oral delivery of insulin using pH-responsive complexation gels. J. Pharm. Sci.

[7] Woitiski CB, Carvalho RA, Ribeiro AJ, Neufeld RJ, Veiga F (2008). Strategies toward the improved oral delivery of insulin nanoparticles via gastrointestinal uptake and translocation. Bio. Drugs.

[8] Des Rieux A, Fievez V, Garinot M, Schneider YJ, Preat V (2006). Nanoparticles as potential oral delivery systems of proteins and vaccines: a mechanistic approach. J. Cont. Rel.

[9] Zhang N, Ping Q, Huang G, Xu W, Cheng Y, Han X (2006). Lectin-modified solid lipid nanoparticles as carriers for oral administration of insulin. Int. J. Pharm.

[10] Rieux A, Fievez V, Garinot M, Schneider YJ, Preat V (2006). Nanoparticles as potential oral delivery systems of proteins and vaccines: A mechanistic approach. J. Cont. Rel.

[11] Ghuman J, Zunszain PA, Petitpas I, Bhattacharya AA, Otagiri M, Curry S (2005). Structural basis of the drug-binding specificity of human serum albumin. J. Mol. Biol.

[12] Kratz F (2008). Albumin as a drug carrier: Design of prodrugs, drug conjugates and nanoparticles. J. Cont. Rel.

[13] Park YJ, Yoon JY, Park HY, Kim JH, Kim WS (1993). Synthesis of model microspheres and adsorption study of bovine albumin. J. Biomed. Eng. Res.

[14] Couvreur P, Barratt G, Fattal E, Legrand P, Vauthier C (2002). Nanocapsule technology: a review. Crit. Rev. Ther. Drug Carr. Syst.

[15] Bajpai AK, Saini R (2005). Preparation and characterization of spongy cryogels of poly (vinyl alcohol) casein system: water sorption and blood compatibility study. Polym. Int.

[16] Bajpai AK, Likhitkar S (2013). Investigation of magnetically enhanced swelling behavior of superparamagnetic starch nanoparticles. Bull. Mater. Sci.

[17] Bajpai AK, Shrivastava M (2001). Water sorption dynamics of hydrophobic ionizable copolymer gels. J. Sci. Ind. Res.

[18] Singh DK, Ray AR (1994). Graft copolymerization of 2-hydroxyethylmethacrylate onto chitosan films and their blood compatibility. J. Appl. Polym. Sci.

[19] Hayes AJ, Markovic B (2002). Toxicity of Australian essential oil Backhousia citriodora (Leman mystlo) Part 1 Antimicrobial Act and In Vitro Cytotoxicity, Food and Chem. Toxic.

[20] Ankarao A, Naik V, Rao KH (2012). Formulation and in vitro evaluation of oral sustained release nanoparticulate delivery system of carvedilol. Internl. J. Res. Pharm. Biom. Sci.

[21] Prabhakar S, Bajpai J, Bajpai AK, Tiwari A (2014). Cumulative release of Cifotaxim from interpenetrating networks of poly(vinyl alcohol-G-acrylamide) and chitosan-G-polyacrylamide chains. Polym. Bull.

[22] Chairam S, Somsook E (2008). Starch vermicelli template for synthesis of magnetic iron oxide nanoclusters. J. Magn. Magn. Mater.

[23] Bakkialakshmi S, Barani V (2013). FTIR study on the interaction of quercetin and amantadine with egg albumin. Inter. J. Pharm. Chem. Bio. Sci.

[24] Sharifi S, Behzadi S, Laurent S, Forrest ML, Stroeve P, Mahmoudi M (2012). Toxicity of nanomaterials. Chem. Soc. Rev.

[25] Quintana JR, Valderruten NE, Katime I (1999). Synthesis and swelling kinetics of poly(dimethylaminoethyl acrylate methyl chloride quaternary-co-itaconic acid) hydrogels. Langmuir.

[26] Liang Y, Deng L, Chen C (2011). Preparation and properties of thermoreversible hydrogels based on methoxy poly (ethylene glycol) – grafted chitosan nanoparticles for drug delivery systems. Carbohydr. Polym.

[27] Rao KSVK, Naidu BVK, Subha MCS, Sairam M, Aminabhavi TM (2006). Novel chitosan based pH-sensitive interpenetrating network for the controlled release of Cefadroxil. Carbohydr. Polym.

[28] Bajpai AK, Bhanu S (2007). Dynamics of controlled release of heparin from swellable crosslinked starch microspheres. J. Mater. Sci. Mater. Med.

[29] Tavakol M, Vasheghani-Farahani E, Dolatabadi-Farahani T, Hashemi-Najafabadi S (2009). Sulfasalazine release from alginate- N, O carboxymethyl chitosan gel beads coated by Chitosan. Carbohydr. Polym.

[30] Chouhan R, Bajpai AK (2010). Release dynamics of ciprofloxacin from swellable nanocarriers of poly (2-hydroxyethyl methacrylate): an in vitro study. Nanomed. Nanotechnol. Boil. Med.

[31] Pourjavadi A, Mahdavinia GR (2006). Superabsorbency, pH-sensitivity and swelling kinetics of partially hydrolysed Chitosan-g-poly (acrylamide) Hydrogels. J. Turk. Chem.

[32] Bajpai AK, Bundela H (2009). Development of poly(acrylamide)-hydroxyapatite composites as bone substitutes: Study of mechanical and blood compatible behavior. Polym. Compos.

[33] Yoon JY, Kim JH, Kim WS (1999). The relationship of interaction forces in the protein adsorption onto polymeric microspheres. Colloids. Surf. A.

